# Effect of family-centered care interventions on well-being of caregivers of children with cerebral palsy: a systematic review

**DOI:** 10.12688/f1000research.133314.1

**Published:** 2023-07-06

**Authors:** Deepalaxmi Paresh Poojari, Shashikiran Umakanth, G. Arun Maiya, Bhamini Krishna Rao, Sonia Khurana, Senthil Kumaran D, Radhika Attal, Marie Brien

**Affiliations:** 1 Department of Physiotherapy, Manipal College of Health Professions, Manipal Academy of Higher Education, Manipal, Karnataka, 576104, India; 2 Department of Medicine, Dr TMA Pai Hospital, Udupi, Manipal Academy of Higher Education, Manipal, Karnataka, 576101, India; 3 Department of Physical therapy, College of Health Sciences, Old Dominion University, Norfolk, Virginia, USA; 4 Enabling Inclusion Program, Amar Seva Sangam, Ayikudi, Tamil Nadu, India

**Keywords:** Developmental disability, family participation, Parent well-being, Physical health, Mental health, Parent-professional partnership, Parent education, Collaborative care

## Abstract

**Background**: Caring for a child with long-term functional limitations can have a negative impact on the physical and psychological well-being of the caregiver. Family-centered care (FCC) interventions have the potential to empower caregivers and contribute to their well-being. This systematic review aimed to synthesize existing evidence on the effectiveness of FCC interventions in improving the well-being of caregivers of children with cerebral palsy (CP), and identify the key components of such interventions that are most commonly practiced and deemed effective.

**Methods**: This review systematically searched seven databases for randomized controlled trials that evaluated the effectiveness of any FCC intervention on the well-being of caregivers of children with or at risk of CP. We used the Cochrane RoB 2.0 tool to assess risk of bias and Critical Appraisal Skills Programme (CASP) checklist for critical appraisal. Due to high heterogeneity of studies, narrative synthesis was used to summarize the data.

**Results**: The review consists of 11 studies which were categorized into five sections based on the components of FCC intervention provided in each individual study: 1. Information provision, and Enabling and partnership (n= 5); 2. Information provision, and Respectful and supportive care (n= 1); 3. Enabling and partnership (n= 2); 4. Enabling and partnership, and Respectful and supportive care (n= 2); 5. Information provision, Enabling and partnership and Respectful and supportive care (n= 1). Risk of bias was low in four studies, unclear in two studies, and high in five studies.

**Conclusion**: FCC interventions were found to be effective in improving caregivers’ satisfaction with attainment of child and caregiver goals. Evidence from multiple studies does not strongly support the effectiveness of FCC interventions on caregiver’s mental health, parenting and personal outcomes. Limited evidence precludes a conclusion on the effectiveness of the components of FCC on well-being of caregivers of children with CP.

## Introduction

Family-centered care (FCC) approach holds fundamental importance to professional practice that conveys dignity and respect to families, information provision for informed decision-making, consideration of the family’s preferences and priorities, and collaborative partnerships between the provider and family. ^1^ This approach may enable caregiver access to various healthcare services through education and counseling, ^2^ support groups, ^3^ information about their child’s condition, ^4^ skill training, ^5^ involvement in setting goals for their children, ^6^ or establishing a strong caregiver-professional partnership.

Children with cerebral palsy (CP) may have a range of impairments that limit their daily activities such as mobility, self-care, communication, and participation, requiring special care services. ^7^^,^^8^ Apart from their daily duties, caregivers take up multiple roles such as handling the medical, rehabilitation, and financial services, in an attempt to provide the best care for their child. Hence, caring for a child with CP demands adjustment in the caregiver’s lifestyle based on the child’s needs and impacts the caregiver’s personal, family, social, and financial well-being. ^9^^,^^10^^,^^11^ Caring for a child with long-term functional limitations may affect the physical as well as the psychological well-being of the caregiver. ^12^^,^^13^ Parents nurturing a child with CP often experience isolation, anxiety, and depression. ^14^^,^^15^ Therefore, meeting the informational, resources, emotional, social, and monetary needs of the caregiver would be crucial to reduce their burden. ^16^^,^^17^ Moreover, since children with CP require long-term multidisciplinary care, providing a continuum of care through a family-centered approach may be able to reduce the caregiver burden, enhance their capacities and empower them to care for their children. ^1^^,^^5^ This will help improve health consequences for both children and their caregivers as well as facilitate their active participation in the community. ^18^

A review of systematic reviews on family-centered care interventions by Park *et al.* (2018), ^4^ provided evidence of the benefits of family-centered care interventions on patients, families and healthcare professionals. However, this study pertained to varied patient populations. A systematic review of family-centered care for children with special healthcare needs by Kuhlthau *et al.* (2011) also found positive effects on health, family function and impact, satisfaction, and communication. ^2^ However, there is a dearth of literature assessing the effectiveness of family-centered care interventions on the well-being of caregivers of children with CP. A comprehensive synthesis of the effectiveness of FCC is essential to provide reliable evidence to practitioners, researchers, and policymakers for the development of strategies for the implementation of care, and hence pave the way for the effective delivery of services to the CP community. Hence, the primary objective of this review was to synthesize evidence on the effectiveness of family-centered care interventions on the well-being of caregivers of children with CP. Realizing the importance of families as a resource in care delivery, it is crucial to identify the best way of empowering them, meeting their needs, and incorporating their participation in therapy. Therefore, our secondary objective was to identify the components of family-centered intervention that are commonly practiced and deemed to be most effective for caregiver well-being.

## Methods

### Protocol registration

This systematic review was conducted in accordance with the preferred reporting items for systematic reviews and meta-analyses 2020 (PRISMA) guidelines. ^19^ The systematic review protocol was prospectively registered with PROSPERO (No. CRD42021233854) and can be accessed at https://www.crd.york.ac.uk/prospero/display_record.php?ID=CRD42021233854.

### Search strategy

Seven databases - Cochrane, Pubmed, Scopus, CINAHL Plus, EMBASE, Web of Science, and ProQuest - were searched from inception to 30 ^th^ September 2022. A systematic search strategy (dataset 1 in Extended data) was used employing the PICO format using filters - Human and English. ^20^ Furthermore, we examined the reference list of the included articles to identify any relevant articles for this review.

### Eligibility criteria

Randomized controlled trials (RCTs) that assessed the effectiveness of the Family-centered approach on caregiver well-being, and conformed to our inclusion criteria were included. The population was limited to primary caregivers of age above 18 years, providing care for children with or at risk of CP at any severity (any level of GMFCS) and up to 18 years. However, studies that included caregivers who are not the primary caregiver of the child, with a diagnosed psychiatric illness, or have children with any other physical disability were excluded. Articles with children with multiple disabilities were excluded if authors failed to provide information and sub-group analysis for children with CP and their caregivers within two weeks of email request. Any intervention which is family driven or has the core components of family-centered care were included: Respectful and supportive care - Social or peer support groups; Information provision - Information sharing, caregiver education through direct education employing online presentation or guiding manual or web-based education, caregiver skill training, caregiver instructions; Co-ordinated and comprehensive care - Interdisciplinary communication, multidisciplinary approach or rehabilitation; Enabling and partnership - collaborative relationship with the caregivers, collaborative goal setting, joint/shared decision making, activity selection, ongoing evaluation, parent-professional partnership, parental advocacy. ^18^ The last component ‘General information’ was not included as a part of FCC in this review as it often forms a part of usual care. Studies comparing the intervention to standard practice such as regular care advised by the paediatrician or other health professionals applied in any setting were included. Studies that reported any well-being outcomes related to caregivers such as quality of life, physical health and fitness, psychological health, satisfaction, family empowerment, adaptation, burden, and level of knowledge were primarily included. Secondary outcomes evaluating the health and well-being of children with CP were also noted but were not necessary for inclusion. All non-human studies and those not in the English language were excluded.

### Data screening and extraction

Data screening and selection were done using Rayyan software (alternative to Covidence or DistillerSR). Two reviewers (DP and RA) independently performed title and abstract, and full-text screening on Rayyan software. Any discord between the two reviewers was settled by consensus. If disagreement persisted, it was settled by team discussions with other researchers in this review (SK, SKD). Independent double data extraction was performed by two reviewers (DP and RA) using a data collection form prepared on Microsoft Word, and discrepancies were handled via discussions. The following data were extracted from each study: Basic study details- author, setting, study design, year of publication; sample size, eligibility criteria; Characteristics of caregivers - age, sex, education, occupation, type of family, socio-demographic details; Characteristics of children with CP - age, sex, type of CP, GMFCS level, MACS level; Intervention details using TIDieR checklist; Results of outcomes - outcome measures, time points, statistical analysis methods such as measures of mean, median, SD, interquartile range, confidence interval, effect size, p-value, and missing information. If effect size was not reported, wherever possible Cohen’s d was calculated. ^21^ Other methods were also used to calculate effect size from odds ratio and median values. ^22^^,^^23^ The corresponding authors were contacted for any missing or unclear information.

As there is a large disparity amongst the included studies in type and dose of intervention, and outcome measures, conducting a meta-analysis is not valid. Therefore, narrative synthesis was chosen to answer the objectives of this review. We classified the studies based on the components of family-centered care (as discussed under eligibility criteria) reflected in their interventions. An intervention may include more than one FCC component. Therefore, for the purpose of synthesis, studies with similar combinations of FCC domains will be combined, compared, and contrasted.

### Quality assessment

To assess the quality of the included studies, two reviewers (DP and RA) independently scored the risk of bias using the Cochrane ‘Risk of Bias 2’ (RoB 2) tool for randomized trials. ^24^ Disagreements between reviewers were resolved through discussion or expert advice from a third reviewer (SK or SKD). Authors of studies were directly contacted if the target information was unreported or unclear. The studies were summarized as having low risk, some concern, or high risk of bias. For critically appraising the RCTs, CASP Randomised Controlled Trial Standard Checklist ^25^ was scored independently by two reviewers (DP and RA). Any disagreements were solved via discussions or by involving a third reviewer (SK or SKD). No scoring system was used as recommended by the CASP checklist developers.

A traffic light system was used to categorize the effectiveness of different outcome domains across the studies to summarize the effectiveness of FCC interventions on caregiver well-being and infant outcomes. Moderate to large effect sizes in a low/some concern risk of bias study were coded green. Small effect sizes in a low/some concerns risk of bias study, or moderate and large effect sizes in a high risk of bias study were coded yellow. No or negative effect was indicated via red colour. The green, yellow and red colour coding indicate advice for implementing the intervention in clinical practice as- effective, use with caution, and not effective respectively.

## Results

**Figure 1.**
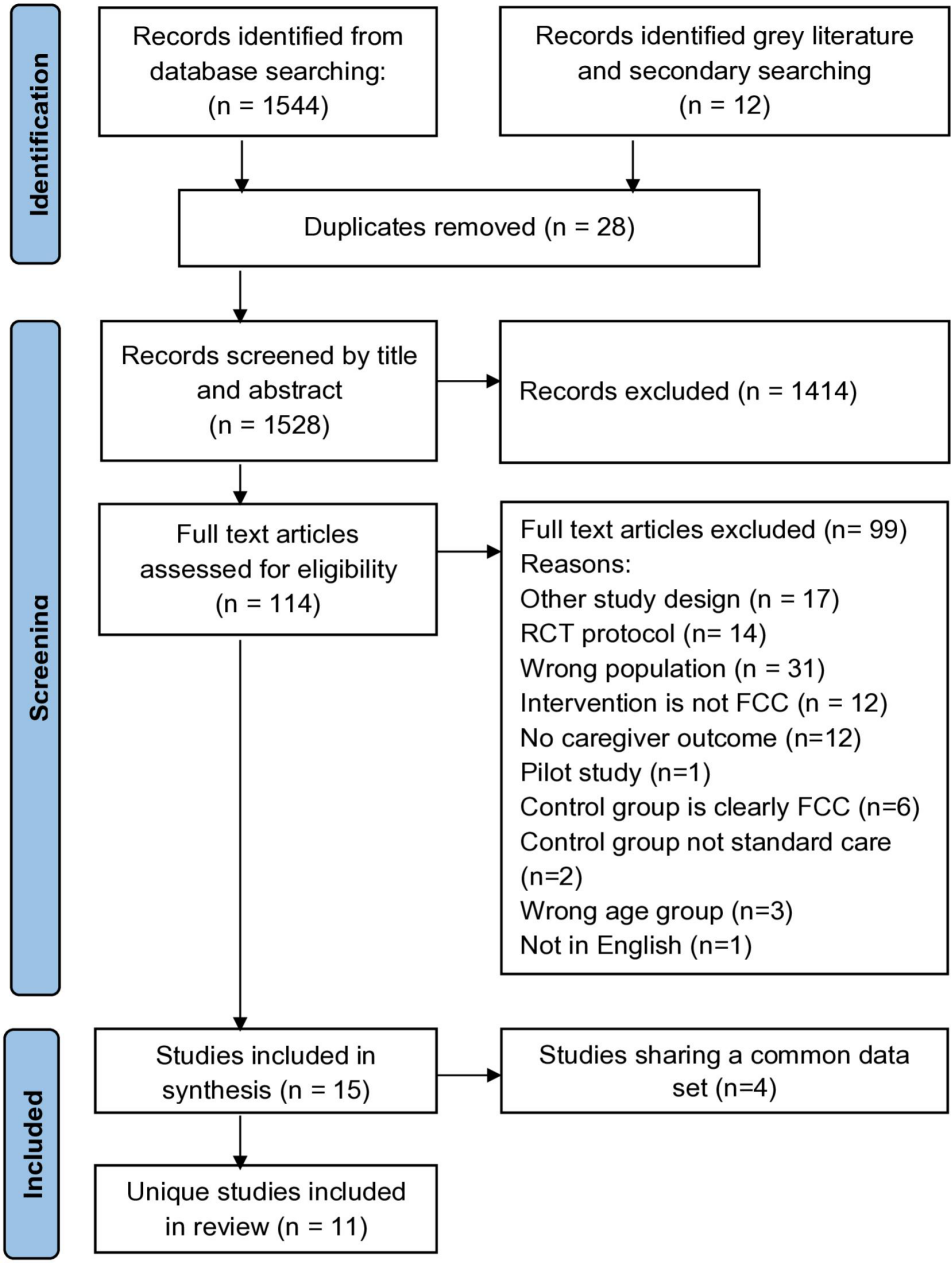
PRISMA 2020 flow diagram. 11 unique articles are included in this synthesis.

The database search yielded 1,544 studies, and an additional 12 articles were found through a secondary search. After removing 28 duplicates, 1,528 studies were screened for title and abstract eligibility, with 1,414 studies getting excluded. 114 articles underwent full-text examination, of which 99 articles were excluded for various reasons reported below. Out of the 15 articles included, n=7 studies were published as a single paper, and n=4 studies were published as eight papers. Therefore, 11 unique articles are included in this synthesis. The PRISMA Flow chart in Figure 1 depicts the results of the search process, and the PRISMA 2020 checklist is provided in data set 2 in Extended data.

### Study characteristics

All the studies are randomized controlled trials, of which nine studies involved children with CP, ^6^^,^^26^^–^^33^ one study involved infants at high risk of CP, ^34^ and lastly, one study involved both children diagnosed or at high risk of CP. ^35^ Three studies were located in Australia, ^29^^,^^32^^,^^34^ and one each in the United Kingdom, ^33^ Tanzania, ^26^ Netherlands, ^35^ Norway, ^31^ Brazil, ^28^ Iran, ^6^ Canada, ^30^ and Denmark. ^27^ In two studies, ^29^^,^^32^ the interventions directly targeted the caregivers while in the remaining studies, the interventions targeted the caregiver indirectly by focussing on improvement in child-related outcomes. The sample size in these studies varied from n= 21 to n= 118, for the parents and children with CP. Table 1 provides an overview of the characteristics of each study, while the TIDieR checklist (Extended data, dataset 3) details the interventions used in each trial. There was a lot of heterogeneity in the focus of interventions. Various caregiver well-being outcomes such as family needs, mental health, empowerment, parenting, satisfaction, quality of life, perception of family centeredness, or caregiver assistance, and child outcomes such as feeding, behaviour, motor or function were included. Dataset 4 in Extended data summarizes the description of outcome measures and intervention effectiveness.

**Table 1.** Characteristics of the included studies.

Author, year, Country	Sample Size	Participants (Children with CP) Age in years Mean (SD) or median (IQR) Sex in %	Participants (Caregivers) Age in years Mean (SD) or median (IQR)	Intervention group		Control group
Weindling *et al.*, 2007 ^33^ United Kingdom	N=88 Intervention 1: 31 Intervention 2: 28 Control: 29	Children with spastic CP < 4 years FSWG: 21.2 ± 9.2 months, 59% male PAG: 19.3 ± 8.7 months, 57% males Control: 18.9 ± 8.7 months, 68% males	Caregivers of children with CP Maternal age: 30.9 ± 0.2 years Paternal age 34.4 ± 6.6 years	Intervention 1: Family support worker group (FSWG): Standard physiotherapy + Family support worker to discuss family needs and provide support	Intervention 2: Physiotherapy assistant group (PAG): Extra Physiotherapy along with standard physiotherapy to increase dose of intervention	Standard Physiotherapy (mainly NDT)
Mlinda *et al.*, 2018 ^26^ Tanzania	N= 118 Intervention: 69 Control: 49	Children < 5 years with moderate-to-severe CP Intervention: 28.5 (12.3) months, 46.0% males Control: 28.9 (13.0) months, 48.9% males	Caregivers of children with CP Intervention: 30.3 ± 5.2 years Control: 31.5 ± 5.34 years	Nutrition education and training on feeding and positioning skills		Usual Care
Whittingham *et al.*, 2014 ^32^ Secondary study: Whittingham *et al.*, 2016 ^39^ Australia	N= 67 Intervention 1: 22 Intervention 2: 23 Control: 22	Children with CP, 2– 12 years of age, GMFCS level I-V SSTP: 5.45 ± 3.16 years, 59.1 % boys SSTP + ACT: 5.52 ± 3.17 years, 73.9 % boys Waitlist: 4.96 ± 2.95 years, 59.1% boys	Parents of children with CP who self-identified the need for a parenting intervention SSTP: 38.67 (5.55) years SSTP + ACT: 37.88 (9.39) years Waitlist: 39.65 (6.09) years	Intervention 1: Stepping Stones Triple P (SSTP) Parenting Interventions for targeting behavioural and emotional problems in childhood	Intervention 2: Stepping Stones Triple P and Acceptance and Commitment Therapy (SSTP + ACT) ACT: Cognitive behavioural therapy for improving psychological flexibility to handle behavioural problems	Waitlist After postintervention assessment, SSTP was offered.
Whittingham *et al.*, 2022 ^29^ Australia	N= 67 Intervention: 37 Control: 30	Child with CP, between 2-10 years Intervention: 5 years 8 months ± 2.36, 59% males Waitlist: 5 years 6 months ± 2.60, 87% males	Parents of children with CP Age not reported	Immediate Parenting Acceptance and Commitment Therapy (PACT) intervention via an online course to improve psychological flexibility to parent this population		Waitlist control
Hielkema *et al.*, 2020 ^35^ Secondary study: Hielkema *et al.*, 2020 ^37^ Netherlands	N=43 Intervention: 23 Control: 20	Infants at very high-risk of CP before 9 months CA Intervention: 1.4 (0.7–2.8) months, 65% males Control: 2.5 (1.8–4.7) months, 55% males	Parents of infants at high risk of CP Intervention: 29 (27–35) years Control: 31 (29–35) years	Coping with and caring for infants with special needs (COPCA) intervention involving parent coaching and adaptive infant motor training		Typical infant physiotherapy (TIP) NDT with a functional approach
Saquetto *et al.*, 2018 ^28^ Brazil	N=60 Intervention: 29 Control: 31	Children with CP between 1–12 years of age. Intervention: 4.66 ± 2.78 years, 41.4% boys Control: 4.52 ± 2.71, 71% boys	Full-time caregiver of child with CP Intervention: 33.38 ± 9.6 years Control: 34.42±10.92 years	Educational programme for primary caregivers to create opportunities for their infants to practice motor control via everyday activities along with conventional rehabilitation		Conventional rehabilitation
Myrhaug *et al.*, 2018 ^31^ Secondary study: Myrhaug *et al.*, 2019 ^38^ Norway	N= 21 Intervention: 11 Control: 10	Children with CP between 3–6 years. All types and functional levels of CP who are eligible for CE courses Intervention: 4 (3–4.5) years, 45% male Control: 4 (3–4) years, 70% males	Parents of children with CP Age not reported	Conductive education (CE) followed by conventional practice		Waiting list: Conventional practice (Functional training)
Kahjoogh *et al.*, 2019 ^6^ Iran	N=30 Intervention: 15 Control: 15	Children with CP, aged 5–11 years with learning capacity GMFCS level I-V Intervention: 6.64 ± 0.97, 46.7% boys Control: Age 7.56 ± 1.59, 66.7% boys	Mothers between 25 and 50 years Intervention: 34.69 ± 4.29 years Control: 38.22 ± 5.98 years	Occupational Performance Coaching (OPC) providing emotional support, information and a structured problem solving process that helped achieve goals along with standard occupational therapy services		Standard occupational therapy services (mainly NDT)
Morgan *et al.*, 2016 ^34^ Australia	N=30 Intervention: 15 Control: 15	Infants aged between 3 and 6 months CA with a diagnosis of CP or at high-risk of CP Intervention: 15.73 ± 4.76 weeks, 53% boys Control: 20.07 ± 5.08 weeks, 60% boys	Mothers of children with CP Intervention: 33.73 (4.73) years Control: 31.07 ± 7.11 years	Goals, Activity and Motor Enrichment (GAME) intervention involving task practice using motor learning strategies delivered along with parent education and involvement.		Standard care
Law *et al.*, 2011 ^30^ Canada	N=91 Intervention: 67 Control: 79	Children between 12 months and 5 years 11 months of age, diagnosed with CP, and all GMFCS levels. Intervention: 3.92 ± 1.42 years, 51% males Control: 3.53 ± 1.43 years, 70% males	Parents of children with CP Age not reported	Context-Focused Approach that involved parents to change constraints in task or environment that hinder child’s performance		Child-Focused Approach: contemporary interventions such as ROM, weight-bearing, etc
Fonvig *et al.*, 2020 ^27^ Secondary study: Rasmussen et al., 2019 ^36^ Denmark	N=60 Intervention: 30 Control: 30	Children with spastic CP, between 5-8 years, GMFCS level I-II Median age: 6 years and 10 months Intervention: Median 6y 6m (2y 8m), 70% boys Control: Median 6y 11m (1y 10 m), 60% boys	Parents of children with CP Age not reported	Individually tailored interdisciplinary intervention based on recommendations from clinical examination as well as an IGA report. Family involvement in planning treatment.		Standard care: Individually tailored interdisciplinary intervention based on clinical examinations without an IGA report.

**Note.** ACT: Acceptance and Commitment Therapy; CA: Corrected age; CE: Conductive education; COPCA: Coping with and Caring for infants with special needs; CP: Cerebral Palsy; FSWG: Family support worker; GAME: Goals, Activity and Motor Enrichment; GMFCS: Gross Motor Function Classification System; IQR: Inter-quartile range; NDT: neurodevelopmental therapy; OPC: Occupational Performance Coaching; PACT: Parenting Acceptance and Commitment Therapy; PAG: physiotherapy assistant group; SSTP: Stepping Stones Triple P; TIP: Typical infant physiotherapy;

### ROB and Quality Assessment

**Figure 2.**
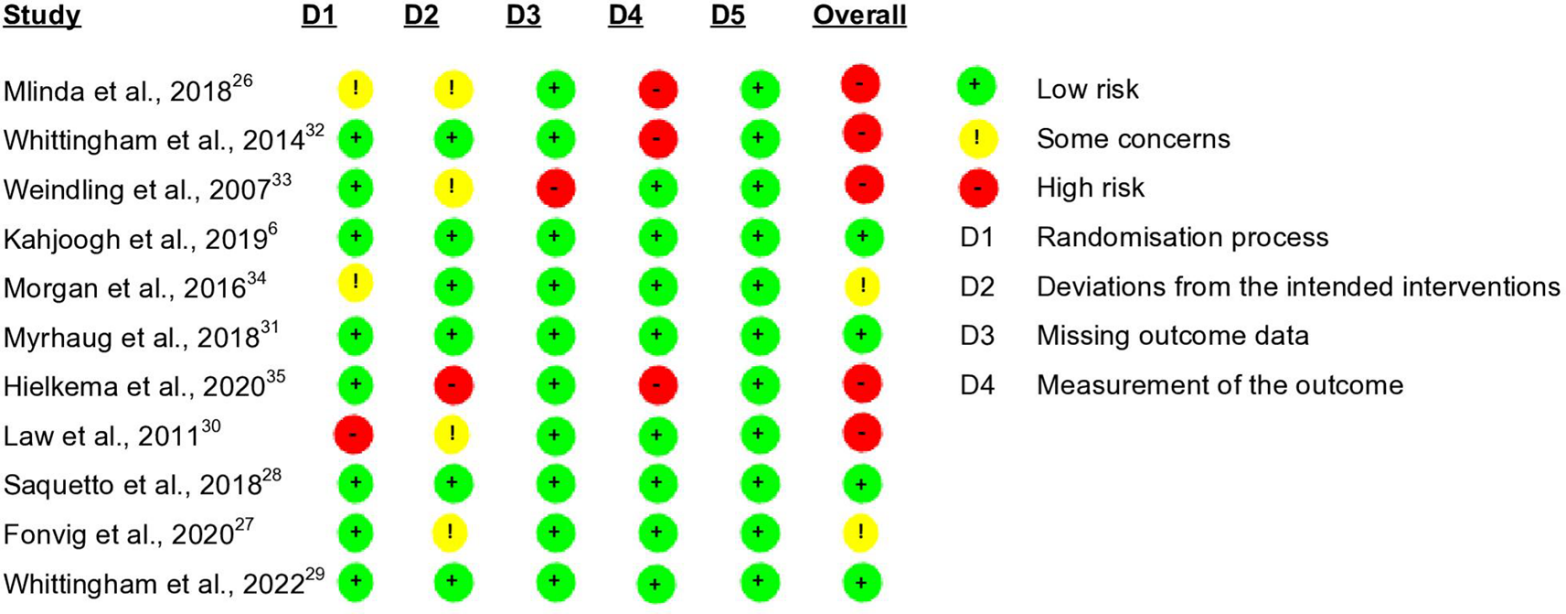
Risk of bias of included studies. Risk of bias was low (green) in four studies, ^6^^,^^28^^,^^29^^,^^31^ unclear (yellow) in two studies, ^27^^,^^34^ and high (red) in five studies. ^26^^,^^30^^,^^32^^,^^33^^,^^35^

According to the Cochrane ROB 2.0 tool for RCTs, four RCTs had low ROB, ^6^^,^^28^^,^^29^^,^^31^ two RCTs had some concerns, ^27^^,^^34^ and five had a high ROB ^26^^,^^30^^,^^32^^,^^33^^,^^35^ ( Figure 2). As our review focussed on caregiver well-being, the majority of caregiver outcomes were patient-reported. Moreover, given the nature of the intervention, the caregivers could not have been blinded to the intervention, and by default that influenced the ROB grading. Therefore, the risk of bias domain assessing outcome assessor’s awareness of intervention and its influence on outcome was rated as ‘probably no’ to avoid categorizing as high-risk of bias on this specific basis. Table 2 represents the results of the CASP Randomised Controlled Trial Standard Checklist. The majority of the studies showed limitations in allocation concealment, investigator and participant blinding, and reporting adverse effects and costs of intervention.

**Table 2.** The results of the critical appraisal using CASP checklist.

Author	Item 1	Item 2	Item 3	Item 4			Item 5	Item 6	Item 7	Item 8	Item 9	Item 10	Item 11
				4A	4B	4C							
Weindling *et al.*, 2007 ^33^	Y	Y	Y	N	N	Y	Y	Y	Y	Y	C	C	C
Mlinda *et al.,* 2018 ^26^	Y	C	Y	N	N	N	Y	C	C	Y	Y	Y	Y
Whittingham *et al.*, 2014 ^32^	Y	Y	Y	N	N	N	Y	Y	Y	Y	Y	Y	C
Whittingham *et al.*, 2022 ^29^	Y	Y	Y	N	N	Y	Y	Y	Y	Y	Y	Y	C
Hielkema *et al.,* 2020 ^35^	Y	Y	N	N	N	Y	Y	Y	Y	Y	C	C	C
Saquetto *et al.*, 2018 ^28^	Y	Y	Y	N	N	Y	Y	Y	Y	Y	Y	Y	Y
Myrhaug *et al.*, 2018 ^31^	Y	Y	Y	N	N	Y	Y	Y	Y	Y	C	C	N
Kahjoogh *et al.*, 2019 ^6^	Y	Y	Y	N	N	Y	Y	Y	Y	Y	Y	C	C
Morgan *et al.,* 2016 ^34^	Y	Y	Y	N	N	Y	C	Y	Y	Y	Y	Y	Y
Law *et al.,* 2011 ^30^	Y	C	Y	N	N	Y	C	Y	Y	N	C	C	C
Fonvig. *et al.,* 2020 ^27^	Y	Y	Y	N	N	Y	Y	Y	Y	Y	C	C	N

Note: Item 1: Clear research question; Item 2: Random assignment of participants to intervention; Item 3: Accounting all participants at conclusion; Item 4: Blinding; 4A: Participant blinding; 4B: Investigator blinding; 4C: Assessor blinding; Item 5: Groups similar at baseline; Item 6: Equal treatment for each study group; Item 7: Intervention effects reported comprehensively; Item 8: Reported precision of the estimate of the intervention effect; Item 9: Benefits of the experimental intervention outweigh the harms and costs; Item 10: Results applicable to your local population; Item 11: Experimental intervention provide greater value to the people in our care than any of the existing interventions; CASP: Critical Appraisal Skills Programme; Y: yes; C: Can't tell; N: No.

**Figure 3.**
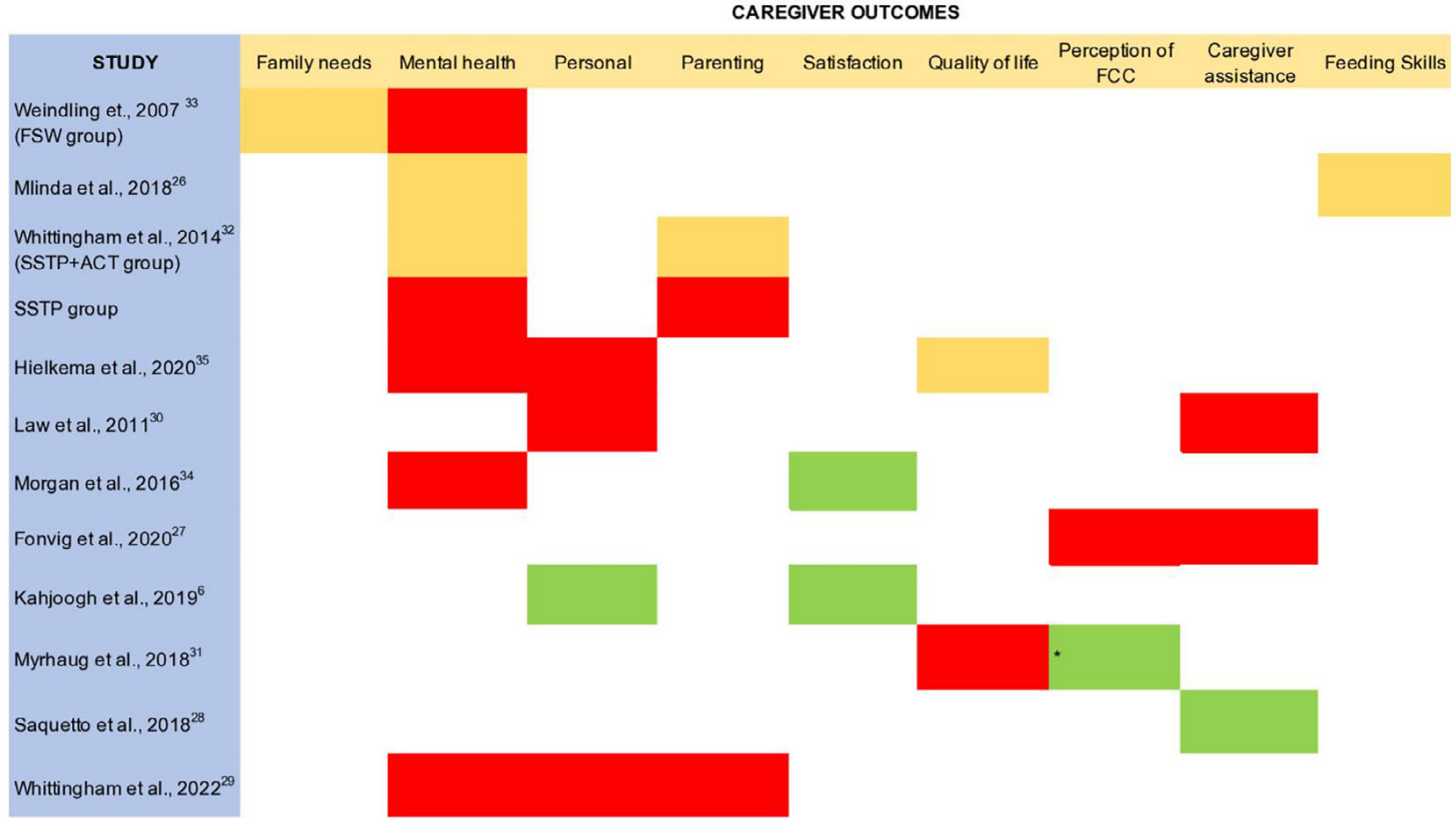
Overall effect of FCC interventions using the traffic light system. Green: moderate to large effect sizes in a low/some concern risk of bias study. Yellow: small effect sizes in a low/some concerns risk of bias study, or moderate and large effect sizes in a high risk of bias study. Red: no or negative effect. *Green on only one domain of Measure of Processes of Care scale (information about child).

Figure 3 demonstrates the overall effect of FCC interventions on caregiver well-being using the traffic light system. FCC interventions are effective (green) in improving caregivers’ satisfaction with attainment of child and caregiver goals. Evidence from a single study indicates that FCC interventions should be used with caution (yellow) to improve family needs and feeding skills. There is inconclusive evidence on the quality of life. Lastly, FCC interventions are not effective (red) to improve caregiver’s mental health, personal outcomes such as empowerment, parenting skills, perception of family centeredness (except information provision about the child), and reduce caregiver assistance in daily activities.

Since the studies include different components of FCC in their interventions, the following section classifies the results based on the core components of family-centered care reflected in the studies. Only two studies incorporated only one FCC component- Enabling and partnership, ^30^^,^^31^ whereas the remaining studies involved multiple FCC components in their intervention. ^26^^–^^29^^,^^32^^–^^35^ The interventions targeted different needs of the caregiver such as social or informational support. Various modes of delivering the intervention such as online or offline platforms, actively through discussions, or passively through mailing child-related information reports were utilized.

### ‘Information provision’ and ‘Enabling and partnership’

Five studies utilized these FCC components in their interventions. ^26^^–^^28^^,^^34^^,^^35^ The results of these papers are also published in two secondary studies from the same sample. ^36^^,^^37^ Two studies, Morgan *et al.* (2016) ^34^ and Hielkema *et al.* (2020) ^35^ involved infants at very high risk of CP ranging from 3 to 9 months corrected age. The intervention in one study ^34^ followed the GAME principles (Goals, Activity and Motor Enrichment) whereas the other study ^35^ followed COPCA principles (Coping with and Caring for infants with special needs). Both studies ^34^^,^^35^ involved active caregiver learning through education and training for the caregivers to become independent in identifying infant’s movements and providing opportunities for motor task practice. Discussions and home programs directed towards a parent-identified goal were given. ^34^ However, the control groups in both studies ^34^^,^^35^ had some family involvement such as parental advice on positioning, handling, feeding, and developmental simulation. ^34^

Improvement was seen in caregiver satisfaction with attainment of goal at 12 months on the COPM (d=0.68). ^34^ Surprisingly, both the studies did not show any improvement in parents’ mental health as measured on Depression Anxiety and Stress Scale (DASS-21) ^34^ and Nijmeegse Ouderlijke Stress Index questionnaire, short version (NOSI-K) ^35^ respectively. However, in the study by Hielkema et al. (2020), significant improvement was seen in caregivers' quality of life (d=0.46) and Infant and Toddler Quality of Life Questionnaire-parent concepts (ITQOL) (impact emotional: d=0.55, impact time: d=0.68) post-intervention. ^35^ No difference was seen in family empowerment and coping mechanisms. With respect to child outcomes, one study showed significant improvement in motor skills at 16 weeks and 12 months on PDMS-2 (d=0.09 and 0.31 respectively), GMFM (d=0.20), cognitive skills at 12 months on BSID-III (d=0.42), and on COPM performance at 16-weeks (d=0.25). ^34^ In contrast, the other study found no significant difference in infant motor, cognitive, behaviour, function outcomes, and quality of life (except general health perceptions d=0.62) as compared to the control group. ^35^

Three studies involved children diagnosed with CP ranging from age 1-12 years. ^26^^–^^28^ All three studies involved caregiver education to facilitate a specific function- improve nutrition and feeding skills, ^26^ become informed about the child’s treatment for gait impairments, ^27^ and facilitate the child’s motor tasks respectively. ^28^ Two studies had active parent involvement via multiple modes of delivering education, ^26^ and caregiver participation in goal setting and practice of functional activities. ^28^ In contrast, the study by Fonvig *et al.* (2020) only provided the information passively by mailing the instrumented gait-analysis report. ^27^ The control group in one study received general health education for parents, ^26^ whereas another study involved multidisciplinary health professionals collaboration. ^27^

The study by Mlinda *et al.* (2018) showed improvement in caregiver feeding skills such as positioning, feeding speed, and feeding support with effect size 0.92, 0.91, 0.69 respectively, and stress (effect size=0.5). ^26^ The study by Fonvig *et al.* (2020) showed no improvement in any domain of the MPOC-20. ^27^ The study by Saquetto *et al.* (2018) showed a large effect in the caregiver assistance required for self-care (ES=5.11) and mobility functions (ES=7.37) on the Pediatric Evaluation of Disability Inventory (PEDI) post-intervention. ^28^ These interventions improved child’s mood (d=0.62), ^26^ gross motor function ( **η** ^**2**^=0.145, large effect), ^27^ and self-care skills (Effect size=2.18, large effect). ^28^

### ‘Information provision’ and ‘Respectful and supportive care’

Only one study by Whittingham *et al.* (2022) ^29^ was included in this category. In this study, an online/telehealth intervention to support the caregivers in positive parenting was delivered using online presentations, activities, and discussion. The intervention showed significant improvement at post-intervention (10 weeks) in non-intrusiveness (d=0.14) and child involvement (d=0.19) on the Emotional Availability Scale, in child involvement (d=0.28) as seen by the Emotional Availability Self Report, and in mindfulness during parenting (d=0.17) using the Interpersonal Mindfulness in Parenting Scale. Parents also reported improvement in acceptance of the child’s CP diagnosis (d=0.64), seeking support (d=0.08), maintaining social connections (d=0.45), and meaningful living (d=0.47) post-intervention and at six months follow up. However, no intervention effect was seen on parent mental health and well-being as measured using the standard outcome measures- the Depression Anxiety and Stress Scale (DASS), the Personal Wellbeing Index (PWI), or the Acceptance and Action Questionnaire (AAQ). With respect to child outcomes, the intervention significantly improved the quality of life in domains of social well-being and acceptance (d=0.08) and participation and physical health subscale (d=0.31) on the Cerebral Palsy Quality of Life scale (CPQOL). However, no intervention effect was seen on child behaviour and adjustment.

### Enabling and partnership

Two studies were found in this category. ^30^^,^^31^ The results of a paper by Myrhaug *et al.* (2018) were also published in one secondary study from the same sample. ^38^ The two studies focussed on children diagnosed with CP utilized collaborative goal setting but differed in the level of parent involvement in therapy. Law *et al.* (2011) ^30^ involved the parents to identify constraints affecting their child's performance. Conversely, in the study by Myrhaug *et al.* in 2018, ^31^ parents were not involved in therapy during the conductive education (CE) courses, while the control group participated in conventional practice with parental involvement.

In the study by Law *et al.* (2011), ^30^ the intervention group showed a small intervention effect on the PEDI Caregiver assistance-mobility sub-scale at 9 months follow-up but no improvement was seen in family empowerment. ^30^ In the study by Myrhaug *et al.* (2018), ^31^ a large effect was seen on receiving more ‘specific information about their child’ on the Measure of Processes of Care scale (MPOC-20) at follow-up (d=1.47). No difference was seen in their global quality of life. With respect to the child outcomes, both the studies did not show any difference in gross motor function, functional skills, preschool participation, and children’s quality of life post-intervention as compared to the control group ^30^^,^^31^^,^^38^

### ‘Enabling and partnership’ and ‘Respectful and supportive care’

Two studies were found in this category. ^32^^,^^33^ The results of a paper were also published in one secondary study from the same sample. ^39^ Both studies provided support to the parents by targeting their needs- needs identified using the family needs scale, ^33^ and positive parenting for child’s behavioural problems. ^32^ The study by Weindling *et al.* (2007) involved joint decision-making between the family support worker and the caregiver to target the needs. ^33^ The study by Whittingham *et al.* (2014) involved Stepping Stones Triple P (SSPT-only) intervention that involved a partnership between the therapist and parent for collaborative goal setting, discussions, and various strategies for positive parenting. ^32^ Another intervention group, SSPT + ACT group (Acceptance and Commitment Therapy) provided additional support to the parents to build their psychological flexibility via goal setting and various exercises. ^32^

The study by Weindling *et al.* (2007) ^33^ did not show improvement in stress (measured on Parent Stress Index) whereas the SSTP+ ACT group in the study by Whittingham *et al.* (2014) ^32^ showed a medium effect in depression (d=0.74) and stress (d=0.79) measured on the Depression Anxiety Stress subscales. Both the studies met caregiver needs as seen by a significant reduction in the Family Needs Scale (FNS) at 18 months follow up (p = 0.001, effect size=–12.0) (but not post-intervention) ^33^ and change in parenting style (reduced overreactivity (d=1.1) and verbosity (d=0.93)). ^32^ The SSTP + ACT group ^32^ showed improvements in child behaviour and emotional problems (ECBI problem d=1.32, ECBI intensity d=0.79, SDQ emotions d=0.16), child hyperactivity (d=0.21), child functional performance in the mobility domain (d=0.03), child quality of life in functioning (d=0.51) and social domains (d=0.64). In the SSTP-only group, ^32^ improvements were seen in child behaviour problems and emotional symptoms. ^32^

### ‘Information provision’, ‘Enabling and partnership’, and ‘Respectful and supportive care’

Only one study was included in this category. ^6^ The study by Kahjoogh *et al.* (2019) ^6^ involved children diagnosed with CP, in the age group of 5-11 years, from all GMFCS levels. Goal setting was done for all caregivers using the COPM (one goal for themselves and two goals for their child) prior to randomization. The caregivers were coached based on the principles of Occupational performance coaching (OPC), from understanding the current scenario, planning actions, analysing performance and problem-solving to achieve the goal. Emotional support was also provided by intentionally listening to the caregivers and providing guidance and encouragement. Information was imparted in consideration of the parent’s experiences as per the principles of OPC. The intervention was given once per week, for 10 weeks or till the goal was achieved. The control group that received conventional therapy, mainly NDT, reported having parent training to move and position their children at home.

The intervention showed a significant and large effect on overall COPM performance and satisfaction scores (η ^2^_p_=0.41 and 0.38 respectively), on individual mother-related performance and satisfaction scores (η ^2^_p_ =0.25 and 0.33 respectively), and on child-related (η ^2^_p_=0.35 and 0.41 respectively) performance and satisfaction scores. Also, a large and significant increase was seen in the caregiver’s self-efficacy (η ^2^_p_ =0.7) measured using the Sherer general self-efficacy scale.

## Discussion

This systematic review aimed to identify the effectiveness of family-centered interventions on the well-being of caregivers of children with CP. We identified 11 unique randomized controlled trials ^6^^,^^26^^–^^35^ to address our objectives. Additionally, we stratified the studies according to the various components of family-centered care in their interventions to identify the impact of these components on the caregiver well-being outcomes. Our review also analysed the effect of FCC interventions on child-related outcomes. We found that family centered care interventions are effective to improve caregivers’ satisfaction with attainment of child and caregiver goals. However, evidence from multiple studies does not strongly support the effectiveness in improving caregiver’s mental health, personal, and parenting skills. Limited evidence in other caregiver outcomes suggests caution in effectiveness of FCC interventions in addressing family needs, and improving feeding skills and quality of life. These results emphasize the need for more interventional studies which are aimed directly at caregivers’ well-being. The following sections will discuss the studies based on the FCC components in their intervention.

### ‘Information provision’ and ‘Enabling and partnership’

The interventions targeting infants at high risk of CP ^34^^,^^35^ allowed caregivers to become informed and actively participate in promoting their infant’s development during daily activities, showing improvement in caregiver satisfaction with goals. The lack of improvement in stress may be because caregivers with CP have a high burden and multiple sources of stress apart from the caregiving responsibilities such as balancing family and work, and financial burdens. ^10^ Moreover, it proved to be successful for infant outcomes in spite of having a greater severely affected population. On the contrary, even though the study by Hielkema *et al.* (2020) ^35^ had a longer intervention duration of one year, no difference was seen in infant outcomes. The influence on caregiver outcomes cannot be relied on as NOSI-K and ITQOL are inappropriate outcome measures for this age group. Moreover, a very small sample size and caregiver dropouts introduced a selection bias underpowering our ability to rely on these findings. With respect to studies on children with CP, all outcomes mentioned in the study by Mlinda *et al.* (2018) ^26^ were binary, non-standard and no clear information was given about them, therefore the results should be interpreted with caution. No improvement in perception of family-centered care was observed in the study by Fonvig *et al.* (2020) ^27^ as the intervention was delivered passively by mail, with no additional help to understand or translate the information to care.

Overall, no improvement was seen in caregiver stress ^34^^,^^35^ except on a non-standardized outcome measure. ^26^ As only one study assessed the effect on quality of life, family empowerment, perception of FCC, caregiver assistance in function, and satisfaction, there is limited evidence to draw a definite conclusion. There is conflicting evidence on the effectiveness of FCC intervention for motor and cognitive outcomes of high-risk infants. ^34^^,^^35^

### ‘Information provision’ and ‘Respectful and supportive care’

The intervention by Whittingham *et al.* (2022) ^29^ modified parent behaviour. The lack of effect on mental health should be considered with caution as parents had normal mental health at baseline. Little improvement was observed in child behaviour problems. Therefore, additional interventions targeting the child behaviour may be necessary in conjunction with parent-focussed interventions. However, as only one study is present in this domain, we cannot conclude if interventions utilizing ‘Information provision’ and ‘Respectful and supportive care’ improved caregiver well-being.

### Enabling and partnership

The two studies incorporated collaborative goal setting with the parents. ^30^^,^^31^ In the study by Law *et al.* (2011) ^30^ both intervention and control groups showed improvement in child outcomes and family empowerment, indicating no additional benefits of context focussed FCC intervention. Conversely, in the study by Myrhaug *et al.* (2018), ^31^ no effect was seen on caregiver’s quality of life and child outcomes. The low sample size, with 50% children belonging to higher disability (GMFCS levels IV and V), and large amounts of conventional therapy in the control group may have diluted the effects. A review on conductive education found inconclusive evidence of its effectiveness due to a lack of quality studies. ^40^ However, positive findings on provision of information for parents on MPOC could be attributed to the availability of many opportunities for informally meeting with the conductor to discuss the child during the conductive education program. Hence, this highlights the power of providing continuous information to the parents about the child’s condition and development during therapy sessions.

Overall, due to varied outcomes, limited evidence exists to draw definitive conclusions on the effectiveness of FCC interventions on caregiver assistance in function activities, family empowerment, perception of FCC, and quality of life. Moreover, no improvement was observed on any child outcomes.

### ‘Enabling and partnership’ and ‘Respectful and supportive care’

The improvement in family needs in the study by Weindling *et al.* (2007) study ^33^ must be accepted with caution due to reduced sample size at 18 months follow-up, especially due to withdrawal of participants with a higher Family Needs score from the intervention group. The family support workers were parents of children with CP who underwent a short training course by a psychologist. They could have identified the caregiver’s needs, however professional support or actively identifying support resources may be required by the parents to meet their enormous and specific needs and actually reduce their stress as seen in a study exploring social support for caregivers of children with chronic diseases. ^41^ The family support workers helped in making the decisions, whereas in the study by Whittingham *et al.* (2014), ^32^ more active support is given to the caregivers such as the practice of beneficial strategies. The improvement in child behaviour and emotional problems may have resulted in reduced depression and stress. Child behavioural problems to be an important predictor for caregivers’ physical and psychological health, and advancement in child behaviour was associated with a better ability to handle stress and higher self-perception. ^7^

Overall, both studies provided support to caregivers, but evidence on the effectiveness of FCC components in reducing stress and depression is conflicting. Only one study showed improvement in child behaviour outcomes and quality of life. ^32^

### ‘Information provision’, ‘Enabling and partnership’, and ‘Respectful and supportive care’

In the study by Kahjoog *et al.* (2019), the specific goals chosen by the caregivers for themselves and the child were addressed by the therapists in the intervention group. ^6^ The intervention process worked in collaboration with the caregivers at all stages-goal setting, analysing performance, and problem-solving to identify treatment solutions. This active involvement of the caregivers in goals chosen by them may have motivated them to work on the goals, and hence, explains the improvement in self-efficacy and COPM scores. Caregivers’ feeling of mastery over a caregiving situation and higher self-esteem predicts better psychological health. ^7^ However, the effect on children's outcomes is unknown. We witness parent involvement in the control group again highlighting their active role in conventional therapy.

As only one study ^6^ was included in this domain, we cannot conclude if interventions utilizing ‘Information provision’, ‘Enabling and partnership’, and ‘Respectful and supportive care’ improved caregiver well-being.

No studies were found that compared the FCC component- ‘Co-ordinated and comprehensive care’ to a control group. The family support worker in addition to physiotherapists in the study by Weindling *et al.* (2007) ^33^ cannot be considered as multidisciplinary care as they are parents of children with CP, not health professionals. It is interesting to observe that five studies involved two to four healthcare professionals working as a team in either intervention or control groups such as physical and occupational therapists ^26^^,^^34^ or neuro-paediatrician, paediatric orthopaedic surgeon, physiotherapist, and a biomechanist. ^27^ This brings to light the established importance of multidisciplinary care for children with CP.

Overall, ten studies incorporated the Enabling and partnership component, ^6^^,^^26^^–^^28^^,^^30^^–^^35^ seven had Information provision, ^6^^,^^26^^–^^29^^,^^34^^,^^35^ and four had a Respectful and supportive care component. ^6^^,^^29^^,^^32^^,^^33^ To address our second objective, Enabling and partnership was the most practiced FCC component. Most family-centered interventions aimed to establish a collaborative relationship with parents, involving them in goal-setting, and capacity-building. These interventions sought to empower the parents to provide independent care for their children, particularly in terms of functional activities at home. However, due to the limited studies and absence of a meta-analysis, we cannot identify which combined FCC components would be most effective for caregiver well-being.

It is interesting to note that even though multiple studies utilized the same FCC component, the delivery of intervention varied. For example, utilizing information materials, discussions, and practical exercises to educate, ^32^ vs. passively mailing the information report to the caregivers. ^27^ Another example would be involving caregivers in goal setting, ^31^ vs. involving them in the therapy activities as well. ^6^ Therefore, the mode of delivering the intervention may also influence the intervention effects.

Research has reported the negative effects of caregiving on carers’ physical health such as fatigue, poor sleep, and musculoskeletal pain. ^42^ However, it is interesting to observe that none of the studies explored the effect of FCC interventions on caregivers’ physical health. This represents a significant gap in the literature and highlights the need for future research to explore this important area.

Conventional therapy cannot be carried out without involving the parents or family. The control group in multiple studies ^6^^,^^26^^,^^31^^,^^34^^,^^35^ also had some degree of parent involvement which could not be excluded as they were a part of the ‘usual care’. Parent involvement varied from education to involvement in therapy sessions or practice of functional activities at home as a home program. We realize that as the primary support system and an essential part of a child’s environment, it is natural for the family to participate in therapy. Moreover, as children with CP require intensive therapy and substantial practice, conventional therapy may have increased parent involvement.

### Strengths and limitations

This review has several strengths and limitations that should be considered. One strength is that we included all studies that reported caregiver outcomes, regardless of whether they also reported infant outcomes. However, studies that only reported infant outcomes without caregiver outcomes were excluded. Therefore, this systematic review does not provide a complete picture of the effect of FCC interventions on infant outcomes. However, this was not our objective and was clearly stated in our eligibility criteria. A meta-analysis could not be conducted due to the heterogeneity of the included studies, and thus we cannot provide a definite summary of the effectiveness of FCC interventions on caregiver well-being. Moreover, as caregiver well-being was our primary outcome, most of the outcomes were participant reported. This introduces some amount of bias as blinding of caregivers is not possible in such cases. Additionally, studies that were not randomized controlled trials, and those published in languages other than English were excluded, which may have resulted in relevant information being missed. However, to the best of our knowledge, only one study was excluded on the basis of language.

### Clinical implications

Core components of FCC that include active interventions are more effective for caregiver well-being than passive interventions. Therefore, healthcare professionals should consider designing interventions that involve active parent engagement. Additionally, providing continuous information to the parents about the child’s condition and development during the therapy sessions is a useful way to deliver information. Thus, health professionals should prioritize educating the parents on their child’s condition, development, and handling during therapy visits to ensure a continuum of updates.

### Future recommendations

Future research should aim to conduct high-quality RCTs with larger sample sizes to better identify the effectiveness of FCC interventions. Future RCTs assessing the effectiveness of FCC interventions need to explore the interventional elements in the control group and clarify the extent of parent involvement. Studies should explore how best they can standardize the control group in an RCT to truly identify the benefits of family-centered interventions. Studies can also explore the effectiveness of different modes of parent participation in family centered care interventions. Further, larger sample sizes are required considering the higher dropout seen in multiple studies. Moreover, future studies should investigate the effect of FCC interventions on caregivers’ physical health and perform a cost analysis to identify the financial burden of these interventions. RCTs should carefully select appropriate outcome measures designed for age and diagnosis, and limit the outcome measures to include the most essential ones to avoid study burden and potential dropouts.

## Conclusion

Despite the many challenges faced by caregivers of children with CP, there are limited FCC interventions that are directly focussed on their well-being. The eleven reviewed studies vary greatly in sample size, interventions focus, dose, theoretical basis, and outcomes, making it difficult to draw concrete conclusions on the effectiveness of FCC interventions on caregiver well-being. However, it can be inferred that FCC interventions are effective in improving caregivers’ satisfaction with attainment of child and caregiver goals. Evidence from multiple studies does not strongly support the effectiveness in improving caregiver’s mental health, personal, and parenting skills. They should be used with caution in addressing family needs, and improving feeding skills and quality of life. Limited evidence and overlap of FCC core components in individual studies precludes a conclusion on the effectiveness of distinct FCC components on the well-being of caregivers of children with CP. However, it is clear that active engagement interventions are more effective for caregiver well-being compared to passive interventions. Establishing active partnerships with caregivers are best to address their needs and priorities.

## Data Availability

No data are associated with this article. Figshare: Search Strategy for “Effect of family centered care interventions on well-being of caregivers of children with cerebral palsy: a systematic review”. Data file 1: Search Strategy. https://doi.org/10.6084/m9.figshare.22344664.v1. ^43^ Figshare: Intervention details using TIDieR checklist for “Effect of family centered care interventions on well-being of caregivers of children with cerebral palsy: a systematic review”. Data file 3: Intervention details using TIDieR checklist. https://doi.org/10.6084/m9.figshare.22344670.v1. ^44^ Figshare: Description of outcome measures and results of intervention effectiveness for “Effect of family centered care interventions on well-being of caregivers of children with cerebral palsy: a systematic review”. Data file 4: Description of outcome measures and results of intervention effectiveness. https://doi.org/10.6084/m9.figshare.22344667.v1. ^45^ Figshare: PRISMA 2020 checklist for “Effect of family centered care interventions on well-being of caregivers of children with cerebral palsy: a systematic review”. Data file 2: PRISMA 2020 checklist. https://doi.org/10.6084/m9.figshare.22344724.v1. ^46^ Data are available under the terms of the Creative Commons Attribution 4.0 International license (CC-BY 4.0).
