# Supplementary material for: Effect of family-centered care interventions on well-being of caregivers of children with cerebral palsy: a systematic review
Source: F1000Res. 2024 May 29;12:790. Originally published 2023 Jul 6. [Version 2] doi: 10.12688/f1000research.133314.2 (PMC11193087; doi:10.12688/f1000research.133314.2)
Supplement: Peer review history

# Reviewer report: Reviewer response for version 2

**DOI:** 10.5256/f1000research.165710.r284090

**Published:** 2024-06-21

**Authors:** Tanya Tripathi

**Affiliations:** 1 Virginia Commonwealth University, The Ohio State University, Columbus, OH, USA

In this revision, the authors have incorporated my previous feedback. I have no further comments.

Are the rationale for, and objectives of, the Systematic Review clearly stated?

Yes

Is the statistical analysis and its interpretation appropriate?

Not applicable

Are sufficient details of the methods and analysis provided to allow replication by others?

Yes

Are the conclusions drawn adequately supported by the results presented in the review?

Yes

Reviewer Expertise:

Physiotherapy and early intervention

I confirm that I have read this submission and believe that I have an appropriate level of expertise to confirm that it is of an acceptable scientific standard.

# Reviewer report: Reviewer response for version 2

**DOI:** 10.5256/f1000research.165710.r284089

**Published:** 2024-06-05

**Authors:** Suruliraj Karthikbabu

**Affiliations:** 1 KMCH College of Physiotherapy, Dr NGP Research and Educational Trust, Coimbatore, Tamil Nadu, India

The revised draft is satisfactory. The authors have incorporated the recommendations and suggestions.

Are the rationale for, and objectives of, the Systematic Review clearly stated?

Yes

Is the statistical analysis and its interpretation appropriate?

Yes

Are sufficient details of the methods and analysis provided to allow replication by others?

Yes

Are the conclusions drawn adequately supported by the results presented in the review?

Yes

Reviewer Expertise:

neurorehabilitation

I confirm that I have read this submission and believe that I have an appropriate level of expertise to confirm that it is of an acceptable scientific standard.

# Reviewer report: Reviewer response for version 1

**DOI:** 10.5256/f1000research.146293.r248360

**Published:** 2024-03-25

**Authors:** Suruliraj Karthikbabu

**Affiliations:** 1 KMCH College of Physiotherapy, Dr NGP Research and Educational Trust, Coimbatore, Tamil Nadu, India

Journal: F1000

- I must appreciate the authors for conducting this important systematic review.
- Overall, the paper is well written.
- I have a few queries and thoughts and please find my review comments below.   
- The authors conducted a systematic review of family-centered care (FCC) interventions on the well-being of caregivers of children with cerebral palsy. They concluded that FCC interventions were effective in improving caregivers’ satisfaction with the attainment of child and caregiver goals, but not for the caregiver’s mental health, parenting and personal outcomes.
- In the introduction, the authors stated how caring for a child with long-term functional disability affects both the physical and psychological well-being of the caregiver, warranting a systematic review to understand the benefits of FCC on multiple variables.
- The authors stated that there is a dearth of literature assessing the FCC on the well-being of caregivers of children with CP. Instead, the authors can elaborate on a diversity of such interventions limiting healthcare professions to choose a specific FCC.
- Search strategies are appropriate and were conducted in seven databases between inception and September 2022. The authors may check if there are any new papers published during the last 18 months. I believe the authors can perform it by the “year” filter and the search-strategy thread.
- Eligibility criteria, data screening and extraction, and quality assessment sections are well explained.
-  “Risk of Bias 2”, CASP checklist and traffic light system are useful and so are the tables and figures. 
- Since caregiver-reported measures are taken in many studies, assessor bias is unavoidable.
- Please share your thoughts on caregivers' burden on looking after younger versus older children with CP. Few studies included infants with CP.   
- Five studies are reporting “information provision’ and ‘enabling and partnership’, providing limited evidence on the said parameters.
- The caregiver well-being domains such as “Information provision’ and ‘Respectful and supportive care’; Enabling and partnership; ‘Enabling and partnership’ and ‘Respectful and supportive care’ and ‘Information provision’, ‘Enabling and partnership’, and ‘Respectful and supportive care’ require more future studies to examine the true benefits of FCC.

Best wishes.

 The reviewer

Are the rationale for, and objectives of, the Systematic Review clearly stated?

Yes

Is the statistical analysis and its interpretation appropriate?

Yes

Are sufficient details of the methods and analysis provided to allow replication by others?

Yes

Are the conclusions drawn adequately supported by the results presented in the review?

Yes

Reviewer Expertise:

neurorehabilitation

I confirm that I have read this submission and believe that I have an appropriate level of expertise to confirm that it is of an acceptable scientific standard.

# Response

**Published:** 2024-04-29

**Authors:** Deepalaxmi Paresh Poojari

**Affiliations:** Physiotherapy, Manipal Academy of Higher Education, Manipal, Karnataka, India

**Response to Reviewers**

**Reviewer one - Dr Tanya Tripathi**

 Comment (C)1. "Firstly, consider tightening the results section to focus specifically on the primary and secondary objectives of the study, which are assessing caregiver well-being and identifying effective intervention components. "

 Response(R)1.Thank you for the suggestion. We have modified our objective statement to state that outcomes for children with CP will also be included, as it is our secondary objective. We have clubbed "Effect on caregiver well-being" and "Components of FCC" as our primary objective and "Effect on children" as a secondary objective. The same has been clarified in our eligibility criteria and limitations. We have highlighted the same in yellow.

 C2."It would be beneficial to include outcome measures and results in Table 1 for each included study." 

 R2. We value your suggestion. However, we will not be able to incorporate outcome measures in Table 1, as there are huge data sets with multiple outcome measures in each study. Please note the outcome measures and results are presented in Data File 4. 

 C3. "Secondly, provide further explanation for the lack of effect on mental health outcomes, considering that parents had normal mental health at baseline. This clarification will help readers better understand the results."

 R3. Thank you for your suggestion. We have incorporated the changes highlighted in green in the manuscript.

 C4. "Lastly, either remove information on child outcomes from the results section, as it was not part of the study's objectives or reword the objectives to include the intention to report on child outcomes. This will enhance the clarity of the study's goals and findings."

 R4. We appreciate your comment and we have clarified it in the objectives. 

**Reviewer two - Dr Suruliraj Karthikbabu**

 Comment (C)1. The authors conducted a systematic review of family-centered care (FCC) interventions on the well-being of caregivers of children with cerebral palsy. They concluded that FCC interventions were effective in improving caregivers’ satisfaction with the attainment of child and caregiver goals, but not for the caregiver’s mental health, parenting and personal outcomes.

 Response (R)1.Thank you for highlighting the important results of the study.

 C2. In the introduction, the authors stated how caring for a child with long-term functional disability affects both the physical and psychological well-being of the caregiver, warranting a systematic review to understand the benefits of FCC on multiple variables. The authors stated that there is a dearth of literature assessing the FCC on the well-being of caregivers of children with CP. Instead, the authors can elaborate on a diversity of such interventions limiting healthcare professions to choose a specific FCC.

 R2. Thank you for your suggestion. We have added the following statement in the introduction:

 ''There is also a scarcity of RCTs which have implemented the Universal model of FCC considering the core components of FCC and there is a need for evidence to understand the specific core components of FCC."

 C3. Search strategies are appropriate and were conducted in seven databases between inception and September 2022. The authors may check if there are any new papers published during the last 18 months. I believe the authors can perform it by the “year” filter and the search-strategy thread.

 R3. Thank you for suggesting this. We have completed an additional review between September 2022 and April 2024 and have included five new articles in the review. All the data sets have been modified accordingly and are highlighted in yellow and the reference DOI has been provided.

 C4. Eligibility criteria, data screening and extraction, and quality assessment sections are well explained.

 R4. Thank you for this acknowledgement.

 C5 “Risk of Bias 2”, CASP checklist and traffic light system are useful and so are the tables and figures. 

 R5. Thank you, we appreciate this recognition of our work.

 C6. Since caregiver-reported measures are taken in many studies, assessor bias is unavoidable.

 R6. Yes, we do agree with this.

 C7.Please share your thoughts on caregivers' burden on looking after younger versus older children with CP

 R7.  In the present study, children with CP were between the ages of 3 months and 16 years. Information needs may have differed for caregivers of younger children, children who had been recently diagnosed with or at risk of CP, or children above 16 years of age who might have gotten used to the entire care system. The craving for child-specific information may be greater in caregivers of younger children than in older children.

 C8. Five studies are reporting “information provision’ and ‘enabling and partnership’, providing limited evidence on the said parameters.

 R8.We agree. Thank you for highlighting this.  Although there are five studies under this domain, the overall effect on caregivers' outcome on standardized outcome was not seen and the child outcome showed conflicting evidence.

 C9.The caregiver well-being domains such as “Information provision’ and ‘Respectful and supportive care’; Enabling and partnership; ‘Enabling and partnership’ and ‘Respectful and supportive care’ and ‘Information provision’, ‘Enabling and partnership’, and ‘Respectful and supportive care’ require more future studies to examine the true benefits of FCC.

 R9. Indeed, the studies had varied ways of providing intervention and sample sizes were not large enough and with high dropouts, thereby hindering conclusive evidence. Overall, five studies fell into the category of high risk. Hence, in order to examine the true benefits of FCC, it is important to have high-quality randomised controlled trials with care in the control groups not overlapping with the intervention groups in terms of the components of FCC received.

# Reviewer report: Reviewer response for version 1

**DOI:** 10.5256/f1000research.146293.r187453

**Published:** 2023-08-31

**Authors:** Tanya Tripathi

**Affiliations:** 1 Virginia Commonwealth University, The Ohio State University, Columbus, OH, USA

This systematic review aimed to assess the effectiveness of family-centered care (FCC) interventions in improving the well-being of caregivers of children with cerebral palsy. The review included 11 studies, which were grouped into five sections based on the components of FCC intervention provided in each study. The review highlights the potential benefits of FCC interventions for caregivers but also underscores the need for further research in this area. Overall, the review is promising, and the methods are scientifically sound. Below are a few suggestions for the authors to improve their reporting of results.

 Firstly, consider tightening the results section to focus specifically on the primary and secondary objectives of the study, which are assessing caregiver well-being and identifying effective intervention components. It would be beneficial to include outcomes measures and results in Table 1 for each included study.

 Secondly, provide further explanation for the lack of effect on mental health outcomes, considering that parents had normal mental health at baseline. This clarification will help readers better understand the results.

 Lastly, either remove information on child outcomes from the results section, as it was not part of the study's objectives, or reword the objectives to include the intention to report on child outcomes. This will enhance the clarity of the study's goals and findings.

Are the rationale for, and objectives of, the Systematic Review clearly stated?

Yes

Is the statistical analysis and its interpretation appropriate?

Not applicable

Are sufficient details of the methods and analysis provided to allow replication by others?

Yes

Are the conclusions drawn adequately supported by the results presented in the review?

Yes

Reviewer Expertise:

Physiotherapy and early intervention

I confirm that I have read this submission and believe that I have an appropriate level of expertise to confirm that it is of an acceptable scientific standard, however I have significant reservations, as outlined above.
